# Rapid in vivo lipid/carbohydrate quantification of single microalgal cell by Raman spectral imaging to reveal salinity-induced starch-to-lipid shift

**DOI:** 10.1186/s13068-016-0691-y

**Published:** 2017-01-03

**Authors:** Liang-da Chiu, Shih-Hsin Ho, Rintaro Shimada, Nan-Qi Ren, Takeaki Ozawa

**Affiliations:** 1 Department of Chemistry, the University of Tokyo, Tokyo, 113-0033 Japan; 2 State Key Laboratory of Urban Water Resource and Environment, School of Municipal and Environmental Engineering, Harbin Institute of Technology, Harbin, 150090 People’s Republic of China

**Keywords:** Raman spectroscopy, Single-cell analysis, Starch–lipid shift

## Abstract

**Background:**

Lipid/carbohydrate content and ratio are extremely important when engineering algal cells for liquid biofuel production. However, conventional methods for such determination and quantification are not only destructive and tedious, but also energy consuming and environment unfriendly. In this study, we first demonstrate that Raman spectroscopy is a clean, fast, and accurate method to simultaneously quantify the lipid/carbohydrate content and ratio in living microalgal cells.

**Results:**

The quantification results of both lipids and carbohydrates obtained by Raman spectroscopy showed a linear correspondence with that obtained by conventional methods, indicating Raman can provide a similar accuracy to conventional methods, with a significantly shorter detection time. Furthermore, the subcellular resolution of Raman spectroscopy enabled not only the concentration mapping of lipid/carbohydrate content in single living cells, but also the evaluation of standard deviation between the biomass accumulation levels of individual algal cells.

**Conclusions:**

In this study, we first demonstrate that Raman spectroscopy can be used for starch quantification in addition to lipid quantification in algal cells. Due to the easiness and non-destructive nature of Raman spectroscopy, it makes a perfect tool for the further study of starch–lipid shift mechanism.

**Electronic supplementary material:**

The online version of this article (doi:10.1186/s13068-016-0691-y) contains supplementary material, which is available to authorized users.

## Background

Microalgae are considered as a solution to many of the environmental problems we face nowadays. They are ideal sources for biofuel production that does not compete for arable land with edible crops, and can grow well under various wastewaters with high uptake rates of enormous pollutants [1, 2]. To enhance their economic feasibility, much effort has been made to optimize the cultivation strategies [3] or to engineer the synthesis pathways of energy-rich metabolites, i.e., lipids and carbohydrates, in microalgae [4], because they are the main materials for the most widely used biofuel products, biodiesel and bioalcohols, respectively [5]. Previous studies have demonstrated that the pathways of lipid and starch synthesis compete for common bio-synthetic precursors [6], and that hyper-accumulation of lipids can be observed when starch synthesis is disrupted in *Chlamydomonas reinhardtii* [7, 8]. Therefore, the rapid detection of lipid/carbohydrate content and ratio in microalgae has become extremely vital when regulating or engineering microalgae for the synthesis of a specific type of biofuel.

Currently, the most widely used technique to quantify the lipid and carbohydrate content in microalgae is gas chromatography (GC) or liquid chromatography (LC) coupled with mass spectrometry (MS). These methods are known to have excellent sensitivity, molecular specificity, and precision, and are the current gold standard for the quantification of cellular compositions in microalgae. However, since the GC/MS or LC/MS methods inevitably involves the chemical extraction of specific compounds (e.g., fatty acid and simple sugars) in the first step, it takes hours of labor work to quantify the lipid/carbohydrate content of a batch of microalgae. Thus, they are considered as destructive, time- and labor-consuming, and environment-unfriendly methods [9]. Also, these conventional methods do not allow the real-time and single-cell monitoring of lipid/carbohydrate content and ratio during cultivation, which limits the scientific conclusion that can be drawn from related studies and hinders their economic impact. To overcome the above shortcomings, Raman spectroscopy has been introduced in recent days as a clean, fast, and non-destructive alternate method to real-time analyze the chemical content in microalgae, especially for the unsaturation degree of lipids [10–12] and starch content [13] in single live cells. In this study, we successfully took a further step to demonstrate that the potential of Raman spectroscopy is not limited to the analysis of single components, e.g., lipid or carbohydrate, separately. Instead, its real impact lies in the simultaneous quantification of multiple components in an easy, fast, and accurate manner.

In this study, we simultaneously visualized the independent behavior of lipid (mainly fatty acids) and carbohydrate accumulation when the microalgal cells experience multiple stress conditions. Our experiment demonstrates that Raman spectroscopic quantification results are comparable to those using conventional methods even when the cells are treated with complex stress conditions that combine multiple stresses with different effects to the cellular composition. Furthermore, since lipid droplets and starch granules are separated structures with comparable size to the focal spot of the Raman excitation light, instead of analyzing the cellular composition by acquiring only one single spectrum from a microalgal cell by laser trapping techniques [10] or flow cytometry techniques [12, 13], we conducted whole cell Raman spectroscopic scans for all presented quantification results. A previous study has shown that such thorough scanning methods can obtain more precise Raman quantification results using a smaller cell number [14]. In addition, we also report high optical resolution Raman images with detailed chemical structures inside microalgal cells that were not seen in previous Raman spectroscopic reports. Our imaging results also showed interesting cellular structures that were not reported before.

## Methods

### Microalgal strain and culture conditions

We chose *Chlamydomonas* sp. as the target of study because it is a widely used as a model organism in biofuel research [3]. The *Chlamydomonas* sp. JSC4 used in this work was isolated from the coast of southern Taiwan. The cells were cultured in modified Bold 6 N medium [15]. 2% CO_2_ was supplied at a rate of 0.1 vvm, and the cells were cultivated at 28 °C with approximately 350 μmol m^−2^ s^−1^ illumination. Since microalgae are known to accumulate starch or lipids under the environmental stress [3], in addition to the control group (N-rich), we treated the microalgal cells with 4 stressful conditions, including 3-day N-depletion, 5-day N-depletion, 5% NaCl+ 3-day N-depletion, and 2% NaCl+ 5-day N-depletion, respectively.

### Conventional lipid/carbohydrate quantification methods

For the quantification of lipid/carbohydrate content, every culture batches that underwent the same treatment were dispensed into two parts, one part for Raman microscopy imaging and the other part for total carbohydrate quantification (*i.e.*, using a colorimetric method with an anthrone reagent) [16] and fatty-acid transesterification coupled with GC/MS quantification [17].

### Raman spectroscopic measurement of standard samples

The Raman microscope we used in this study is a homemade confocal microscopy system with 532 nm continuous-wave laser irradiation. The laser system was Millennia Pro from Spectra-Physics Lasers, Inc. The laser power at the sample was 1 mw. The objective lens used for the study was the CFI S Fluor 40× oil immersion lens with NA 1.30. The spectrometer was the iHR320 model from Horiba, and CCD camera was the liquid nitrogen-cooled Spec-10 2 kV-EV/LN model from Princeton Instruments. The groove density of the grating was 600 grooves/mm and the center wavelength was 500 nm. The spectral resolution of all measurements was 6 cm^−1^.

As for the standard samples that are used to represent the spectra of carbohydrates, lipids, proteins, and carotenoids: starch (from corn), albumin (from bovine serum, fatty acid free), and β-carotene were purchased from Wako Pure Chemical Industries, Ltd. Oleic acid was purchased from Sigma.

### Raman spectral imaging

The same Raman microscope as described above was used for imaging purposes. The typical acquisition time of a single spectrum from a microalgal cell was 1 s. Prior to Raman measurements, *Chlamydomonas* sp. cells cultured in the culture medium were directly plated on MAS-coated glass bottom dishes developed by Matsunami Glass Ind., Ltd. The MAS coating prevented the cells that are attached to the bottom coverslip from floating around. However, the attaching strength was not very strong, so it requires some effort to find cells that have limited movements (most likely because the surface of the microalgal cell wall is round, so the contact area between the cells and MAS-coated surface is limited). During the measurement, the scanning area was decided by the size of the cells, as can be seen in the brightfield imaging mode of the microscope. The scanning area was always set to be larger than the cell to include the whole cell area for analysis. Although the optical resolution of our setup was around 205 nm in the XY plane and 1.8 µm in the Z axis, to reduce the imaging time, we set the scanning steps of all our measurements at 3 steps per micrometer. The acquisition time of the Raman spectral images for the quantification of cellular compositions was around 0.5–1.5 h, depending on the cell size.

### Raman imaging data analysis

The dataset we acquired was a 3D matrix with spatial image as 2 of the axes and spectral information in the 3rd axis. After the hyperspectral Raman imaging dataset was acquired, all obtained spectral images first went through cosmic ray removal, baseline subtraction, singular value decomposition (SVD) denoising, and polynomial fluorescent background subtraction before the spectral content were analyzed. The Raman images of the microalgal cells were generated at this stage by mapping the integrated intensity of the marker bands at each pixels of the image. For the calculation of integrated Raman band intensity, we assumed that all Raman bands are positioned on a slope. Due to the nature of such baseline assumption (assuming peak shoulder as a slope, or baseline fluctuation due to background noise), negative peak intensity values could appear in the Raman images, but we present and calculate only the positive values (as can be seen in Fig. 3, all contrasts start from 0 intensity). When calculating the normalized Raman intensity, we first decided the cell area by counting the number of pixels the microalgal cell span through in the Raman image, summed up the obtained marker band intensities in all pixels, then divided the summed intensity of the respective Raman bands with the number of pixels the algal cell spanned.

## Results and discussion

### Selection of reference Raman peaks for lipid/carbohydrate quantification

**Fig. 1:**
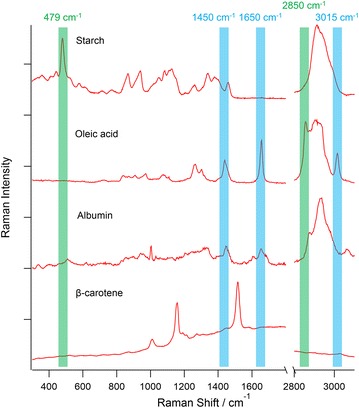
The Raman spectra of starch, oleic acid, albumin, and Raman spectra of starch, oleic acid, albuminna gracilis with stimulated Raman scattering microsc, and carotenoids in algal cells. The 479 and 2850 cm^−1^ Raman bands highlighted by the* green* labels are used for the quantification of starch and lipids (fatty acids), respectively, in this report. The other Raman bands highlighted by the* blue* labels are those that we discuss in the main text but do not choose as the marker band

**Fig. 2:**
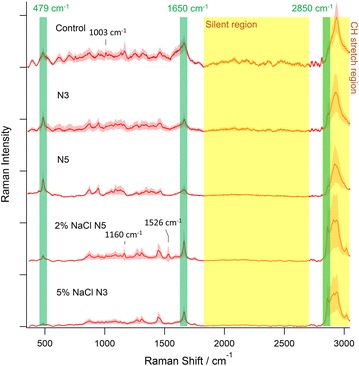
The Raman spectra of control, nitrogen-depleted (3-day nitrogen depleted as N3, 5-day nitrogen depleted as N5), and nitrogen-depleted plus salt-stressed (2% NaCl N5 and 5% NaCl N3) algal cells. The* green* labels highlight Raman bands that we compare between different culturing conditions. The Raman bands that we discuss only in a specific spectrum are indicated by the* black* indicator with their Raman shift value. The* pink* area alongside the spectra represents the spectral standard deviation within a single algal cell

For the quantification of lipid/carbohydrate content and ratio in algae, we first took the Raman spectra of starch, oleic acid, albumin, and β-carotene as the reference for carbohydrate, lipid, protein, and carotenoid spectra in the microalgal cells of *Chlamydomonas* sp., respectively (Fig. 1). Although the amount of carotenoids in microalgal cells is generally not comparable to those of carbohydrates, lipids, and proteins, the reason we include carotenoids in this discussion is because the 532 nm laser we use generates resonance enhancement for the carotenoid Raman spectra in the cells, making their spectra clearly visible under certain conditions (e.g., the 2% NaCl+ 5-day N-depletion-stressed cell in Fig. 2). Since previous studies mostly focused their analysis on either lipids or carbohydrates, such systematic comparison of the Raman spectral profile across the different chemical species in microalgal cells has not been reported before. To maximize the quantification accuracy, it is important to choose the Raman signatures that are strong in the respective chemical species but weak in the others as the reference peak. For example, from the spectra shown in Fig. 1, it is clear that starch has a strong and characteristic C–C–C pyran ring backbone deformation signature at 479 cm^−1^ that are not seen in all other molecular species, making it an ideal marker Raman band for the quantification of starch content in microalgal cells [18].

As for the quantification of lipids (specifically fatty acids, the raw material for biodiesel production), although previous studies tend to use the intensity of 1450 cm^−1^ CH_2_ bend peak to evaluate the total fatty acid content [10, 11], from Fig. 1 it is clear that albumin, a typical protein standard, also possess the same Raman band that peaks at almost the same position with similar band shape, making the band inadequate for fatty acid quantification at low concentrations. Other candidate lipid marker bands include the 1655 and 3015 cm^−1^ signals, but they are both vibrational modes that are related to the C = C double bond in the oleic acid structure, thus cannot serve as a general marker for the lipid content in cells. The best candidate as the fatty acid marker band is therefore the symmetric CH_2_ stretch at 2850 cm^−1^. Although it is positioned on the shoulder of other more dominant CH stretch bands, by assuming its baseline as a slope, it is still possible to extract the intensity information of the 2850 cm^−1^ band alone. A much weaker symmetric CH_2_ stretch mode is also seen in the albumin spectrum in Fig. 1, but since its intensity is barely higher than the baseline slope, and obviously shifted to the higher wavenumber side, it does not influence the lipid quantification as much as the case of the 1450 cm^−1^ CH_2_ bend peak. Furthermore, since the CH_2_ moiety is a highly abundant structure in all fatty acids, the 2850 cm^−1^ Raman signature generally exists in the Raman spectra of all long chain fatty acids [10]. This makes the 2850 cm^−1^ band an ideal marker for the quantification of long-chain fatty acids (e.g., C16-C18). Because the microalgal lipid is mainly composed of fatty acids [3], it should be noted that in this study, whenever lipid is mentioned, we are actually referring to the fatty acid content in the microalgal cells.

To confirm the stability of the Raman measurement, especially the selected carbohydrate and lipid Raman bands, we also conducted a series of time-lapse measurements of standard oleic acid and starch samples every 10 min for continuously 6 h. Since the response of the Raman system is calibrated with standard ethanol sample every day before experiments, we did not perform inter-day verification of the measurement stability. The result showed a 1.7% relative standard deviation for oleic acid measurements and 4.0% relative standard deviation for starch measurements after the fluorescent background from the impurities of the sample was photo bleached out (Additional file 1: Figure S1). We consider such stability as sufficient to generate reliable quantification results inside single living cells.

### Raman spectroscopic analysis of single microalgal cell

**Fig. 3:**
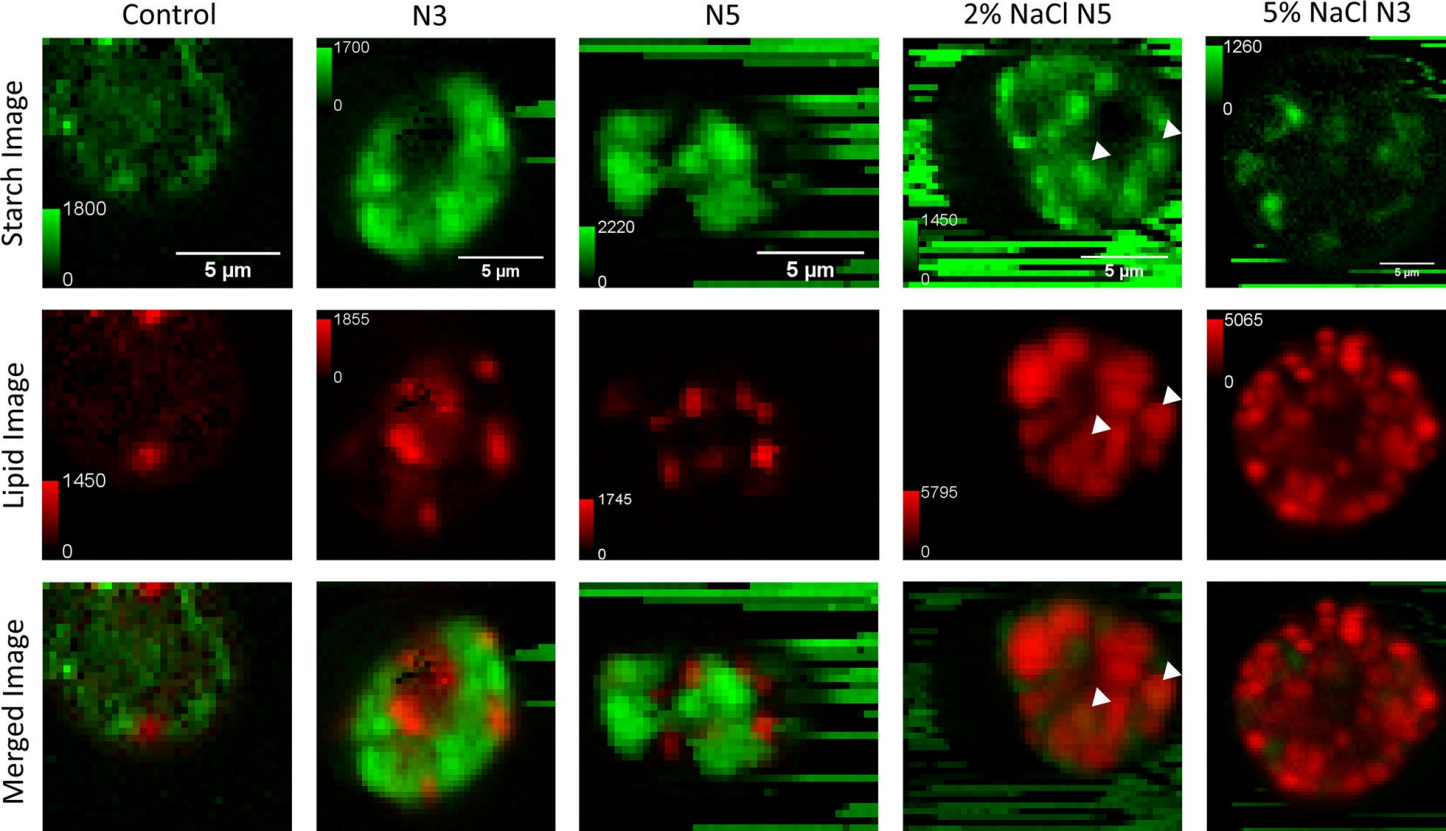
The Raman images of control, N3, N5, 2% NaCl N5, and 5% NaCl N3 algal cells. The *color bar* shows the absolute Raman intensity of the 497 cm^−1^ band for the starch images and the absolute Raman intensity of the 2850 cm^−1^ band for the lipid images. The size of each images are indicated by the *scale bar*. The contrasts for the merged images are normalized to the highest Raman intensity present in the carbohydrate and lipid images of the same cell. The *white arrows* indicate the starch granules that are engulfed by lipid droplets

Having confirmed the marker Raman bands for lipid/carbohydrate quantification, now we focus our discussion on the Raman spectra of the microalgal cells (Fig. 2). The presented spectra were the averaged spectra from the whole mapping results of a single algal cell, and the spectral images of the cells are shown in Fig. 3. An obvious background noise can be immediately recognized in the spectrum of control cell. The spectrum of 3-day N-depleted microalgal cells also shows a similar noise but at a lower intensity. The noise is especially obvious in the silent region of the spectra between 1800 and 2700 cm^−1^, where the spectra of the other microalgal cells are mostly flat. The background noise is mainly due to the auto-fluorescence background in the microalgal cells. Although we have used polynomial fitting to eliminate most of the auto-fluorescence background, the high frequency fluctuation due to white noise and the optical filter transmission spectrum will still remain, resulting in the background fluctuation in the spectra of microalgal cells with strong auto-fluorescence. The raw data without fluorescent background subtraction that shows the absolute auto-fluorescence background are shown in Additional file 1: Figure S2. The different levels of auto-fluorescence background in microalgal cells with different treatments suggest that the pigment composition in microalgal cells changes with the levels of stress. Similar phenomenon was also reported by Masojidek et al. using spectrophotometry [19], which demonstrated that the change in pigment composition is most likely related to the different photosynthesis activity in the control and stressed microalgal cells [19].

In addition to the background fluctuation, Raman spectral differences according to the different treatments-control, N-depletion, and salt addition with N-depletion—can also be seen in Fig. 2. Since we will focus our discussion on starch/-lipid content in later sections, here we first discuss the other chemical information that can be extracted by reading the Raman spectra from microalgal cells. The spectrum of control cells has a broad peak at 1650 cm^−1^ that differs from the sharp oleic acid 1650 cm^−1^ peak in Fig. 1. Moreover, it has the sharpest peak in the CH stretch region (at 2935 cm^−1^) with a slightly steeper slope at the higher wavenumber side, together with a typical phenylalanine band at 1003 cm^−1^. These features highly resemble the albumin spectrum in Fig. 1, demonstrating that the control cells (N-rich cells) have a much richer protein content than the stressed cells, which is in agreement with a previous report [20]. The spectra of 3-day and 5-day N-depletion cells both show a strong and sharp 479 cm^−1^ peak that was assigned as the marker starch band. If we look closer to the spectral shape at the CH stretch region, the spectrum of 5-day N-depletion is the most similar to the standard starch spectrum in Fig. 1, and the spectral shape of 3-day N-depletion condition is between albumin and starch spectrum. These phenomena show that although both N-depletion cells have high starch content, the cells under short-term nitrogen stress would still keep a higher protein ratio, which was also found in other microalgal species (e.g., *Chlorella* sp.) reported via Ho et al. [21]. As for the spectra of cells under both salt addition and N-depletion, their strong and sharp peaks at 2850 and 1650 cm^−1^ resembles to the oleic acid spectrum in Fig. 1. Notably, the spectrum of 2% NaCl+ 5-day N-depletion shows some other interesting features of a weak starch peak at 479 cm^−1^ and an obvious carotenoid spectral feature [22] (Fig. 1) with peaks at 1526 cm^−1^, 1160 cm^−1^. After triplicate analysis, all three cells treated under 2% NaCl+ 5-day N-depletion stress show much stronger carotenoid features than the cells under other treatments. Considering that the spectrum of 5% NaCl+ 3-day N-depletion did not show such carotenoid Raman spectral feature, by testing different culture conditions, it might be possible to specifically enhance the carotenoid production in microalgal cells. Overall, the above results clearly demonstrated that microalga *Chlamydomonas* sp. could produce a relatively high amount of protein content under favorable growth conditions, but would tend to accumulate carbohydrates or lipids when under the conditions of N-depletion or salt plus N-depletion, respectively.

### Raman spectral imaging of microalgal cells

In addition to simple spectroscopic analysis, since our dataset includes the whole hyper spectral images of microalgal cells, we can provide further spatial chemical information that far exceeds what can be obtained from a single spectrum. To focus on the discussions of the lipid/carbohydrate content in microalgal cells, in this study we construct the Raman spectral images using the 479 cm^−1^ carbohydrate Raman band and 2850 cm^−1^ lipid Raman band only. Image reconstruction from the carbohydrate and lipid marker bands in microalgal cells showed aggregated structures that correspond well to the reported starch granule and lipid droplet structures in *Chlamydomonas* sp. [17] (Fig. 3). Some of the starch images display a noisy background with horizontal stripes. The background comes from excreted starch granules that are floating around in the medium. The horizontal stripe pattern is due to the optical trapping of the starch granules by the laser focus; therefore, the trapped granules will stay in the laser focus until they hit obstacles, i.e. microalgal cells in this case. The color contrast between the different cells is not normalized, and the absolute value of the Raman intensity according the contrast is labeled in the color bar. In the merged images, however, we have normalized the carbohydrate and lipid contrast according to their absolute Raman intensity. Starch–lipid shift can be clearly seen by comparing the N-depleted microalgal cells and salt-stressed plus N-depleted microalgal cells. Microalgal cells that are only stressed by N-depletion showed a dominating carbohydrate distribution with a darker lipid contrast, while the adding a stronger salt stress forced the microalgal cell to express a dominating lipid distribution with a darker carbohydrate contrast. Similar lipid and carbohydrate distribution between microalgal cells with the same stress treatments could also be seen by transmission electron microscopy (TEM) (Additional file 1: Figure S3), supporting our observation by Raman spectral imaging. It is worth emphasizing that compared with TEM, Raman spectral imaging has the significant advantage of being capable of keeping the cells alive while quantifying the lipid/starch content, and the Raman signal contrast is proportional to the concentration of the sample. One new finding from the Raman images is that although in most cases, the starch and lipid contrasts do not overlap, few exceptions can be seen (white arrows in the images of 5-day N-depletion and 2% NaCl+ 5-day N-depletion). Since the overlapping structures had around 2 µm diameter, which is even larger than 1.8 µm depth resolution of our Raman imaging setup, it strongly suggests that lipid droplets may actually engulf starch granules in microalgal cells. The TEM image in Additional file 1: Figure S3 also showed a structure similar to lipid droplet engulfed starch granule, further supporting our observation. To the best of our knowledge, this is the first report of discovering such phenomena, and the engulfment process may have some important biological significance.

### Microalgal lipid/carbohydrate quantification by Raman spectroscopy

To better represent the statistical chemical component between different cultured groups, we have also averaged the spectral intensity of the marker carbohydrate (479 cm^−1^) and lipid (2850 cm^−1^) bands from 3 randomly chosen microalgal cells for each experimental condition. Since all cells went through a thorough Raman scan, the spectral sampling size was proportional to the size of microalgal cells, with a total of 2188 spectra for the 3 smallest control microalgal cells, and a total of 6104 spectra for the 3 largest microalgal cells under 5% NaCl+ 3-day N-depletion. Since in conventional methods, the lipid and carbohydrate contents are usually expressed by the percentage of dry cell weight (% DCW), in this study we also normalized the integrated lipid, protein, and carbohydrate Raman intensity from a single algal cell by its cross-sectional area (Table 1). The normalized Raman intensity of each cell was first calculated using all the Raman spectra taken from the cell, and then the statistics of the biomass accumulation level between different cells (Table 1) were calculated. The results are similar to the single cell analyzed in Fig. 2, indicating that N-depleted microalgal cells represented higher carbohydrate content, and salt with N-depletion made the cells show higher lipid content. One interesting observation here is the effect of salt treatment on N-depleted algal cells. It is known that algal cells tend to accumulate starch under mild stress (e.g. N-depletion) and lipid under stronger stress (salt stress) [3]. Our Raman observation clearly demonstrated the more significant starch-to-lipid shift due to stronger salt stress on N-depleted algal cells. Despite the slightly lower level of both the starch and lipid content in 3-day N-depleted cells when compared with 5-day N-depletion, 5% salt stress in 3-day N-depleted cells clearly induced a steeper drop in the starch content and pushed the lipid content higher than the algal cells with 2% NaCl+ 5-days N-depletion. Such highly efficient detection of starch–lipid shift in algal cells within an hour of measurement can never be achieved by conventional isolation techniques combined with mass spectrometry, not to mention if fast averaging methods are applied to quickly obtain the averaged Raman spectrum of a single microalgal cell, the data acquisition time can be suppressed to within seconds [14, 23].

**Table 1:** The averaged “normalized Raman intensity” (total Raman intensity in a cell/number of pixels the cell image cover) and standard deviation of 3 cells for each condition

Cultured conditions	Carbohydrate signal	Lipid signal
Control (N-rich)	226 ± 97	207 ± 83
3-day N-depletion	457 ± 261	525 ± 301
5-day N-depletion	546 ± 167	591 ± 427
2% NaCl with 5-day N-depletion	314 ± 96	1212 ± 386
5% NaCl with 3-day N-depletion	179 ± 45	1376 ± 201

**Fig. 4:**
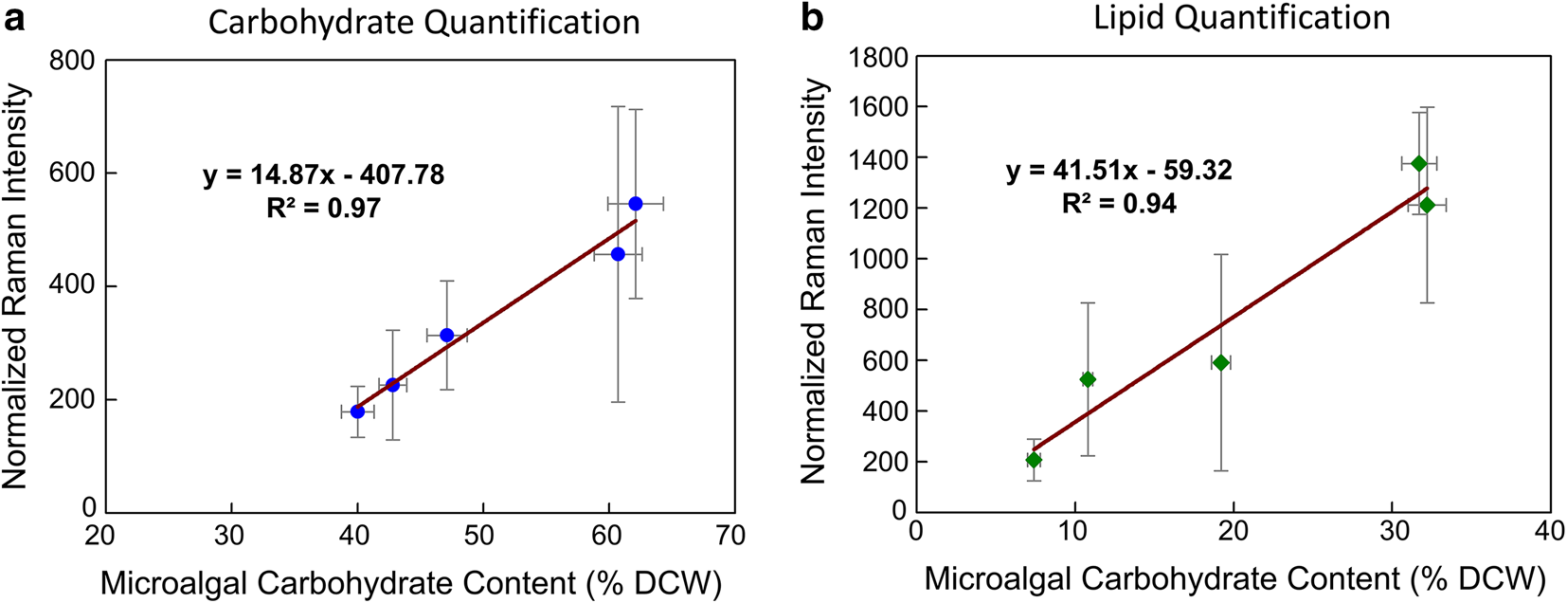
The relationship between Raman measurements and GC/MS measurements on **a** carbohydrate and **b** lipid quantification. The baseline mismatch for carbohydrate quantification is from the excreted starch granules

Eventually, we compared the Raman quantification results to the values obtained via the conventional methods. As shown in Fig. 4, the R^2^ values between the results obtained by the two techniques exceeded 0.9 for both carbohydrate and lipid quantification, indicating that Raman spectroscopy not only has the advantage of real-time carbohydrate/lipid determination of a single microalgal cell, but also has high reliability on quantifying the abundance of carbohydrate and lipid contents simultaneously in microalgal cells. Significant baseline difference can be found when comparing the carbohydrate analysis of the two techniques (Fig. 4a). This is most likely because part of the microalgal cells were disintegrated due to the stress conditions, which resulted in the release of starch granule into the culture medium (Fig. 3). Those suspended starch granules might be mixed into the starch content analysis using conventional methods (because some of the starch granules are hydrophobic, which adhere to algal cells during harvest), but is totally excluded from the Raman measurement. Furthermore, compared with the typically less than 5% standard deviations for conventional methods, a significantly higher standard deviation for the statistical Raman analysis can be seen. Since our setup stability test also showed a less than 5% standard deviation for the continuous Raman measurement of standard samples (Additional file 1: Figure S1), such high standard deviation is unlikely due to the instability of our Raman setup. Furthermore, such significant deviation between the biomass accumulation levels of independent microalgal cells was reported from multiple research groups working on the analysis of cellular components at single-cell level [12, 24]. The standard deviation difference between the Raman and the conventional measurements most likely reflects the actual deviation at the single-cell and batch statistics levels. In other words, for cells under the same batch culture conditions, their collective biomass accumulation behavior is rather stable, as shown in many conventional analysis studies. However, at the single-cell level, each cell could actually behave very differently from the others. This further addresses the importance of single microalgal cells analysis in addition to batch analysis for the optimization of biofuel production.

## Conclusions

In this study, we have demonstrated that Raman spectroscopy can serve as a fast, easy, and reliable method to simultaneously quantify the abundance of carbohydrate and lipids in single microalgal cells. Reducing the labor intensive isolation procedures in conventional quantification methods will also lead to less experimental errors in the long run. The Raman imaging of stressed algae has also revealed the existence of lipid droplet engulfed starch granules, which, to the best of our knowledge, has not been reported in the relevant studies. Different from the conventional isolation and mass spectrometry techniques, the non-destructive nature of Raman spectroscopy will allow the time-lapse Raman imaging study of microalgal cells, which is expected to further elucidate the detailed accumulation process and starch–lipid shift mechanism of carbohydrate or lipids when the microalgal cells are grown under stress conditions. Moreover, our Raman measurement demonstrated that the individual algal cells could accumulate different levels of carbohydrate or lipids even under the same environmental stress. There could be many reasons behind such inhomogeneity, such as the different growth cycle between cells, genetic or epigenetic reasons, or even division of labor between individual microalgal cells within the same population. Thus, Raman spectroscopy will serve as a novel technique to identify the actual cause of such individual difference between the biomass accumulation processes of individual microalgal cells. From a more industrial point of view, our study indicates that Raman spectroscopy can be applied to the selection of microalgal cells that are either suitable for bioethanol or biodiesel production. Further breeding of the selected strains will create more efficient biofuel sources at single-cell level. Taken together, we believe that the label-free and non-destructive real-time analysis of carbohydrate and lipid contents within a single microalgal cells by Raman spectroscopy has enormous potential in both microalgal-based biofuel research and related industry application in the near future.

## Additional file

**Additional file 1: Figure S1**. The stability test of our Raman setup over 6 hour’s measurement. **Figure S2.** The raw data without fluorescence background subtraction calculations for the data shown in Fig. 2. **Figure S3.** The TEM images of microalgal cells under different stress conditions.

## Abbreviations

GC: gas chromatography
LC: liquid chromatography
MS: mass spectrometry
SVD: singular value decomposition
TEM: transmission electron microscopy

## References

[1] Jones CS, Mayfield SP (2012). Algae biofuels: versatility for the future of bioenergy. Curr Opin Biotechnol.

[2] Razzak SA, Hossain MM, Lucky RA, Bassi AS, de Lasa H (2013). Integrated CO_2_ capture, wastewater treatment and biofuel production by microalgae culturing—a review. Renew Sustain Energy Rev.

[3] Ho S-H, Ye X, Hasunuma T, Chang J-S, Kondo A (2014). Perspectives on engineering strategies for improving biofuel production from microalgae—a critical review. Biotechnol Adv.

[4] Trentacoste EM, Shrestha RP, Smith SR, Gle C, Hartmann AC, Hildebrand M, Gerwick WH (2013). Metabolic engineering of lipid catabolism increases microalgal lipid accumulation without compromising growth. Proc Natl Acad Sci.

[5] Jegannathan KR, Chan E-S, Ravindra P (2009). Harnessing biofuels: a global Renaissance in energy production?. Renew Sustain Energy Rev.

[6] Li Y, Han D, Yoon K, Zhu S, Sommerfeld M, Hu Q, Richmond A, Hu Q (2013). Molecular and cellular mechanisms for lipid synthesis and accumulation in microalgae: biotechnological implications. Handbook of microalgal culture.

[7] Li Y, Han D, Hu G, Dauvillee D, Sommerfeld M, Ball S, Hu Q (2010). Chlamydomonas starchless mutant defective in ADP-glucose pyrophosphorylase hyper-accumulates triacylglycerol. Metab Eng.

[8] Li Y, Han D, Hu G, Sommerfeld M, Hu Q (2010). Inhibition of starch synthesis results in overproduction of lipids in Chlamydomonas reinhardtii. Biotechnol Bioeng.

[9] Ho SH, Chen CY, Chang JS (2012). Effect of light intensity and nitrogen starvation on CO_2_ fixation and lipid/carbohydrate production of an indigenous microalga Scenedesmus obliquus CNW-N. Bioresour Technol.

[10] Wu H, Volponi JV, Oliver AE, Parikh AN (2011). Simmons B a, Singh S: in vivo lipidomics using single-cell Raman spectroscopy. Proc Natl Acad Sci USA.

[11] Hosokawa M, Ando M, Mukai S, Osada K, Yoshino T, Hamaguchi H-O, Tanaka T (2014). In vivo live cell imaging for the quantitative monitoring of lipids by using Raman microspectroscopy. Anal Chem.

[12] Wang T, Ji Y, Wang Y, Jia J, Li J, Huang S, Han D, Hu Q, Huang WE, Xu J (2014). Quantitative dynamics of triacylglycerol accumulation in microalgae populations at single-cell resolution revealed by Raman microspectroscopy. Biotechnol Biofuels.

[13] Ji Y, He Y, Cui Y, Wang T, Wang Y, Li Y, Huang WE, Xu J (2014). Raman spectroscopy provides a rapid, non-invasive method for quantitation of starch in live, unicellular microalgae. Biotechnol J.

[14] Schie IW, Kiselev R, Krafft C, Popp J (2016). Rapid acquisition of mean Raman spectra of eukaryotic cells for a robust single cell classification. Analyst.

[15] Berges JA, Franklin DJ, Harrison PJ (2001). Evolution of an artificial seawater medium: improvements in enriched seawater, artificial water over the last two decades. J Phycol.

[16] Choi SP, Nguyen MT, Sim SJ (2010). Enzymatic pretreatment of *Chlamydomonas reinhardtii* biomass for ethanol production. Bioresour Technol.

[17] Ho S-H, Nakanishi A, Ye X, Chang J-S, Chen C-Y, Hasunuma T, Kondo A (2015). Dynamic metabolic profiling of the marine microalga *Chlamydomonas* sp. JSC4 and enhancing its oil production by optimizing light intensity. Biotechnol Biofuels.

[18] Liu Y, Xu Y, Yan Y, Hu D, Yang L, Shen R (2015). Application of Raman spectroscopy in structure analysis and crystallinity calculation of corn starch. Starch - Stärke.

[19] Masojídek J, Torzillo G, Kopecký J, Koblížek M, Nidiaci L, Komenda J, Lukavská A, Sacchi A (2000). Changes in chlorophyll fluorescence quenching and pigment composition in the green alga *Chlorococcum* sp. grown under nitrogen deficiency and salinity stress. J App Phycol..

[20] Huo Y-X, Cho KM, Rivera JGL, Monte E, Shen CR, Yan Y, Liao JC (2011). Conversion of proteins into biofuels by engineering nitrogen flux. Nat Biotechnol.

[21] Ho S-H, Huang S-W, Chen C-Y, Hasunuma T, Kondo A, Chang J-S (2013). Bioethanol production using carbohydrate-rich microalgae biomass as feedstock. Bioresour Technol.

[22] Hashimoto A, Yamaguchi Y, Chiu L, Morimoto C, Fujita K, Takedachi M, Kawata S, Murakami S, Tamiya E (2015). Time-lapse Raman imaging of osteoblast differentiation. Sci Rep.

[23] Okuno M, Hamaguchi H (2010). Multifocus confocal Raman microspectroscopy for fast multimode vibrational imaging of living cells. Opt Lett.

[24] Wakisaka Y, Suzuki Y, Iwata O, Nakashima A, Ito T, Hirose M, Domon R, Sugawara M, Tsumura N, Watarai H, Shimobaba T, Suzuki K, Goda K, Ozeki Y (2016). Probing the metabolic heterogeneity of live Euglena gracilis with stimulated Raman scattering microscopy. Nat Microbiol.

