# Supplementary material for: Rapid in vivo lipid/carbohydrate quantification of single microalgal cell by Raman spectral imaging to reveal salinity-induced starch-to-lipid shift
Source: Biotechnol Biofuels. 2017 Jan 3;10:9. doi: 10.1186/s13068-016-0691-y (PMC5210293; doi:10.1186/s13068-016-0691-y)
Supplement: Supplementary file 1 — Additional file 1: Figure S1. The stability test of our Raman setup over 6 hour’s measurement. Figure S2. The raw data without fluorescence background subtraction calculations for the data shown in Fig. 2. Figure S3. The TEM images of microalgal cells under different stress conditions. [file 13068_2016_691_MOESM1_ESM.pdf]

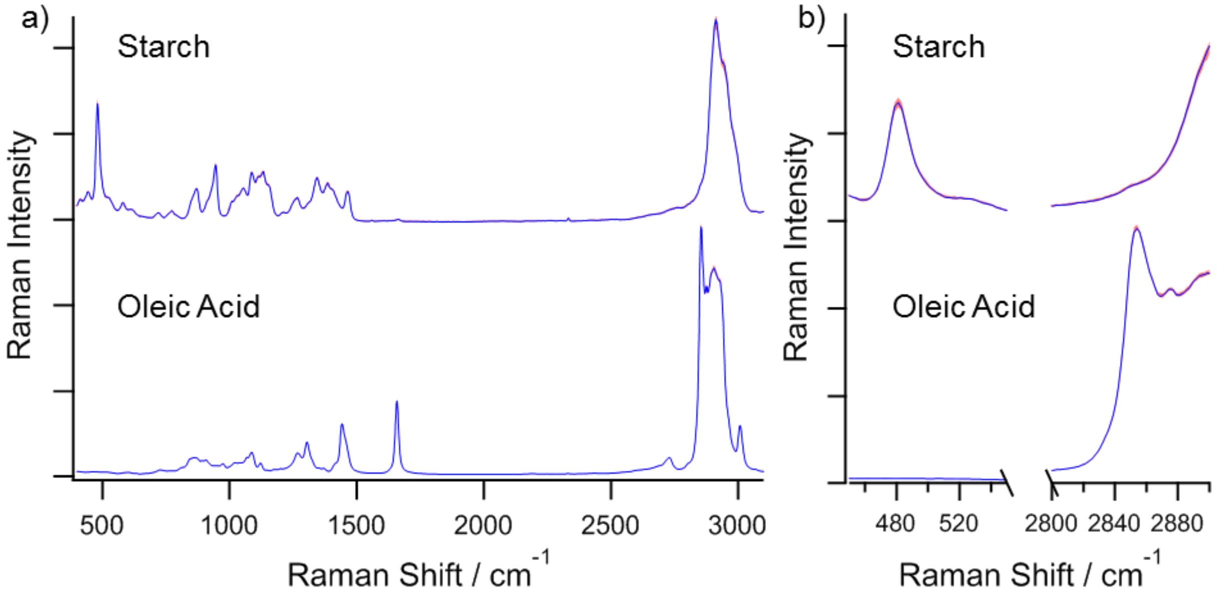

Sup. 1. The averaged spectra (blue line) and their standard deviation (red area alongside the spectra) of 36 time-lapse measurements of standard starch and oleic acid samples over 6 hours. a) The whole range starch and oleic spectra. b) The close-up display of the 479 cm^{-1} marker band for carbohydrates and 2850 cm^{-1} marker band for lipids (fatty acids) in a).

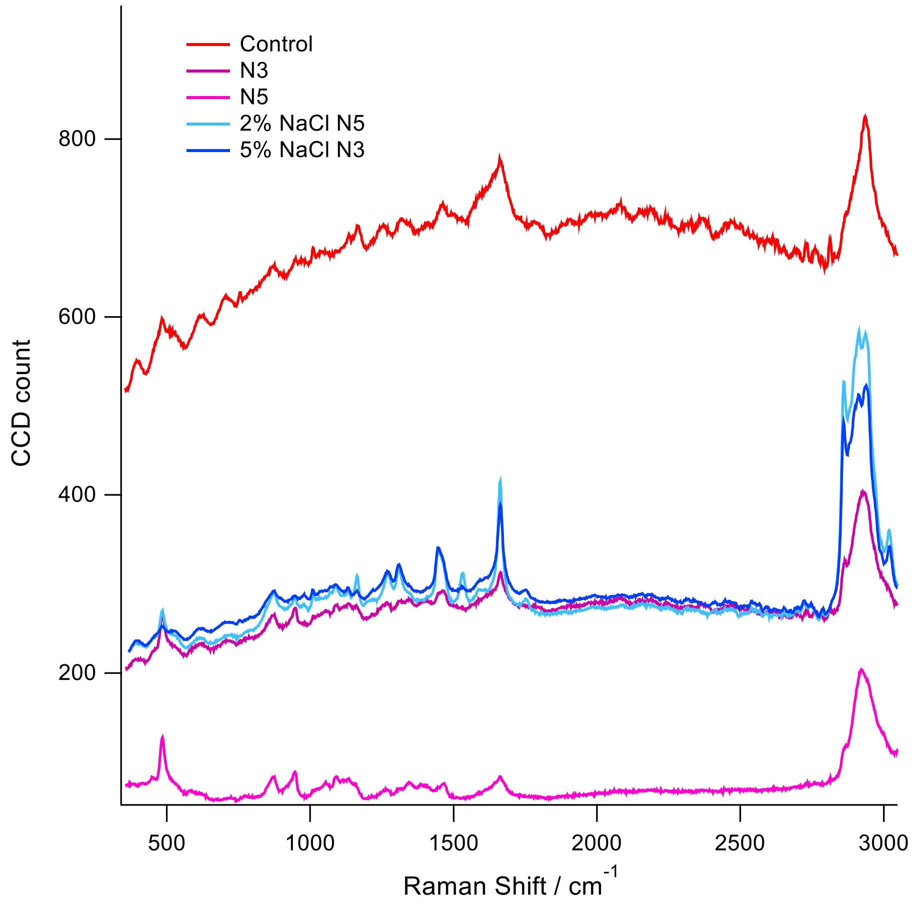

Sup. 2. The raw data without fluorescence background subtraction calculations for the data shown in Fig. 2. Notice that the y axis for all 5 spectra are the absolute CCD count in this figure. No offsets or magnifications are applied in the y axes of the 5 displayed spectra, which is different from the case in Fig. 2.

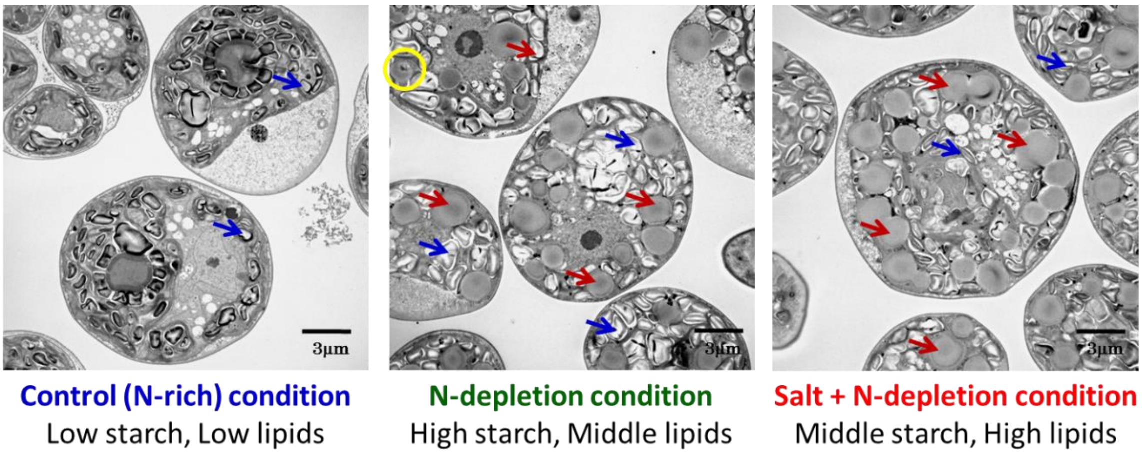

Sup. 3. The energy storage compound of algal cells shifted from starch to lipid upon salinity stress through TEM micrographs (Blue arrow- starch granule; Red arrow- oil drops). The yellow circle highlights the structure that seems to be a lipid droplet engulfed starch granule.
